# Optimizing remote ischemic conditioning for acute ischemic stroke: a systematic review and meta-analysis of treatment duration and reperfusion strategies

**DOI:** 10.3389/fneur.2026.1787078

**Published:** 2026-05-19

**Authors:** Yushuo Liu, Minjuan Zhang, Qihui Yang, Fan Yang

**Affiliations:** School of Physical Education, Yunnan Normal University, Kunming, Yunnan, China

**Keywords:** ischemic stroke, meta-analysis, neuroprotection, remote ischemic conditioning, reperfusion injury, thrombectomy

## Abstract

**Background:**

Recent large-scale randomized controlled trials (RCTs) regarding remote ischemic conditioning (RIC) for acute ischemic stroke (AIS) have yielded inconsistent results. We conducted a systematic review and meta-analysis to evaluate the efficacy and safety of RIC, with a focused analysis on the impact of treatment duration and reperfusion strategies.

**Methods:**

We searched PubMed, Embase, the Cochrane Library, and Web of Science from inception to March 30, 2026, for eligible RCTs comparing RIC with control in AIS patients; reference-list screening and supplementary Google Scholar searches were also performed. The primary outcome was functional independence (Modified Rankin Scale score 0–2) at 90 days. The secondary outcome was 90-day mortality. Exploratory subgroup analyses were performed according to treatment duration (longer-duration vs. shorter-duration) and reperfusion context (endovascular thrombectomy [EVT], intravenous thrombolysis [IVT], and non-reperfusion/mixed standard-care contexts). Risk Ratios (RRs) were calculated using a random-effects model.

**Results:**

A total of 12 RCTs involving 5,301 patients were included. Overall, RIC was associated with a modest but statistically significant increase in the rate of functional independence at 90 days (RR 1.05, 95% CI 1.01–1.09; *p* = 0.01). Exploratory subgroup analyses suggested that longer-duration RIC protocols (≥5 days) may be associated with more favorable outcomes (RR 1.08), whereas shorter-duration protocols did not show a clear signal of benefit (RR 1.03). Similarly, a potential benefit was observed in patients undergoing EVT (RR 1.25, 95% CI 1.07–1.46; *p* = 0.005), whereas no additional benefit was evident in patients receiving IVT alone (RR 0.99; *p* = 0.82). No significant difference in 90-day mortality was observed between groups (RR 0.99; *p* = 0.94). Sensitivity analyses were broadly consistent with the primary result; however, exclusion of the largest trial attenuated the pooled effect and rendered it no longer statistically significant.

**Conclusion:**

RIC appears to be a safe adjunctive therapy for AIS and may offer a modest functional benefit. The available evidence suggests, but does not prove, that longer treatment regimens and use in patients undergoing mechanical thrombectomy may be associated with more favorable outcomes. These subgroup findings should be interpreted cautiously and confirmed in future high-quality trials.

## Introduction

Acute ischemic stroke (AIS) remains a leading cause of mortality and long-term disability worldwide. While reperfusion therapies, specifically intravenous thrombolysis (IVT) and mechanical thrombectomy (MT), have revolutionized stroke management (1), a significant “treatment gap” persists. A substantial proportion of patients fail to achieve functional independence despite successful vessel recanalization, often due to ischemia–reperfusion injury (IRI), microvascular no-reflow, and delayed neuronal death (2). Consequently, there is an urgent need for adjunctive neuroprotective strategies that can extend the therapeutic time window or enhance the efficacy of reperfusion.

Remote ischemic conditioning (RIC) represents a novel, non-invasive strategy designed to induce endogenous neuroprotection (3). By applying transient, sublethal ischemia to a distant limb (typically via a blood pressure cuff), RIC triggers a systemic protective cascade. Preclinical models have consistently demonstrated that RIC can reduce infarct size and improve neurological outcomes by modulating inflammatory responses, stabilizing the blood–brain barrier, and improving collateral circulation via humoral and neural pathways (4, 5).

Despite the solid biological plausibility and promising Phase II results, the translation of RIC into clinical practice has been hindered by conflicting results from large-scale Phase III trials. The landmark RICAMIS trial (Remote Ischemic Conditioning for Acute Moderate Ischemic Stroke, 2022), which enrolled over 1800 patients in China, provided compelling evidence that a longer-duration RIC protocol (daily treatment for 10–14 days) significantly improved functional outcomes at 90 days (6). Conversely, the more recent large-scale RESIST trial (Remote Ischemic Conditioning in Stroke, 2023) reported neutral results, casting doubt on the routine utility of RIC in the ambulance or pre-hospital setting (7).

A critical examination of these divergent trials reveals substantial heterogeneity in treatment duration (longer-duration daily sessions vs. single pre-hospital session) and reperfusion strategies (IVT, MT, or conservative management). Recent post-hoc analyses of the RESIST trial (8) and the newly published SERIC-EVT trial (9) suggest that specific subgroups, particularly those undergoing endovascular treatment, may derive significant benefit. Whether the efficacy of RIC is dose-dependent (requiring repeated stimuli) or substrate-dependent (requiring vessel recanalization to deliver humoral factors) remains an unresolved debate.

Therefore, we conducted this systematic review and meta-analysis to synthesize the most up-to-date evidence, including pivotal RCTs published in 2025 (8–10). Importantly, this study was designed not simply as a repetition of previous meta-analyses, but as an updated and more clinically focused evaluation of RIC in imaging-confirmed acute ischemic stroke (AIS). Compared with the comprehensive meta-analysis by Kan et al. (38), which included a broader spectrum of ischemic cerebrovascular conditions and emphasized protocol-level optimization, our review specifically focuses on contemporary AIS populations and examines whether treatment duration and reperfusion strategy may modify the observed treatment effect. Our specific objective was to evaluate the efficacy and safety of RIC in AIS patients, with a focused analysis on optimizing treatment duration and exploring the potential interaction with reperfusion strategies. Specifically, the incremental value of the present review lies in incorporating the latest 2025 randomized evidence and in examining whether treatment duration and reperfusion context may help explain the discrepant findings across contemporary AIS trials.

## Methods

### Protocol and registration

This systematic review and meta-analysis was conducted in accordance with the Preferred Reporting Items for Systematic Reviews and Meta-Analyses (PRISMA) 2020 statement (11). The study protocol, eligibility criteria, outcomes, and subgroup analyses were defined *a priori* and followed throughout the review process to minimize selection bias. However, the review was not prospectively registered in PROSPERO. We acknowledge this as a methodological limitation and state it explicitly here for transparency.

### Search strategy

We systematically searched PubMed, Embase, the Cochrane Library, and Web of Science for eligible randomized controlled trials (RCTs) from inception to March 30, 2026. The search strategy combined medical subject headings (MeSH, where applicable) and free-text terms related to “Remote Ischemic Conditioning,” “Ischemic Stroke,” “Cerebral Ischemia,” “Reperfusion,” and “Thrombectomy.” To improve comprehensiveness, we also performed supplementary Google Scholar searches and manually screened the reference lists of relevant systematic reviews (12) and all eligible articles for additional trials. No language restrictions were applied. The updated search identified no additional eligible studies for inclusion in the quantitative synthesis.

### Inclusion and exclusion criteria

Studies were included if they met the following PICOS criteria:

1. Population: adult patients (≥18 years) diagnosed with acute ischemic stroke (AIS) confirmed by neuroimaging (CT or MRI).
2. Intervention: remote ischemic conditioning (RIC) applied via limb ischemia–reperfusion cycles (e.g., using blood pressure cuffs) initiated within the acute phase of stroke.
3. Comparison: sham conditioning (simulated cuff inflation) or standard medical care alone.
4. Outcomes: the primary outcome was functional independence, defined as a Modified Rankin Scale (mRS) score of 0–2 at 90 days. Secondary outcomes included mortality within 90 days (safety endpoint) and adverse events related to RIC.
5. Study design: randomized controlled trials (RCTs).

We excluded studies involving patients with peripheral arterial disease (PAD), chronic ischemia, hemorrhagic stroke, or transient ischemic attacks (TIA). Animal studies, observational studies, and trials with insufficient data for effect size estimation were also excluded.

### Data extraction and quality assessment

Two independent reviewers screened titles, abstracts, and full-text articles. Data were extracted using a standardized data extraction form, including study characteristics (author, year, country), sample size, baseline demographics (age, sex, NIHSS score), intervention protocols (cycle duration, number of cycles, treatment days), reperfusion therapies (IVT, EVT, or none), and outcome data. Disagreements were resolved by consensus or consultation with a third reviewer.

The methodological quality of the included RCTs was assessed using the Cochrane Collaboration’s Risk of Bias tool (13). Seven domains were evaluated: random sequence generation, allocation concealment, blinding of participants and personnel, blinding of outcome assessment, incomplete outcome data, selective reporting, and other sources of bias. Each domain was graded as “low risk,” “high risk,” or “unclear risk”.

### Statistical analysis

Statistical analyses were performed primarily using Review Manager (RevMan) version 5.4.1 (14), and additional sensitivity and publication-bias analyses were conducted in R (version 4.5.2) using the meta package. For dichotomous outcomes (functional independence and mortality), Risk Ratios (RRs) with 95% confidence intervals (CIs) were calculated using the Mantel–Haenszel method. Given the potential clinical and methodological heterogeneity across trials (e.g., varying RIC protocols, stroke severities, and healthcare settings), a random-effects model was employed for all analyses to provide a conservative estimate.

Heterogeneity was assessed using the Cochrane *Q* test and quantified with the *I*^2^ statistic; *I*^2^ values of 25%, 50%, and 75% were considered to indicate low, moderate, and high heterogeneity, respectively (15).

Pre-specified subgroup analyses were conducted to explore the sources of heterogeneity based on: (1) Treatment Duration (Longer-duration [repeated daily sessions for ≥5 days] vs. Shorter-duration [single session or <5 days]); and (2) Reperfusion Strategy (EVT, IVT, or non-reperfusion/mixed standard-care contexts). For the EVT subgroup, data were specifically extracted from trials or subgroups of trials that enrolled patients undergoing mechanical thrombectomy (8, 9).

Sensitivity analyses were conducted for the primary outcome using a leave-one-out approach and by excluding studies with non-standard outcome definitions (16), sub-study/*post hoc* reports (8), and the largest individual trial (6). A stricter sensitivity analysis restricted to 90-day mRS 0–2 studies was also performed.

Publication bias for the primary outcome was assessed by visual inspection of funnel plots and formally evaluated using Egger’s linear regression test and Begg’s rank correlation test. A two-sided *p*-value < 0.05 was considered statistically significant.

## Results

### Study selection

**Figure 1:**
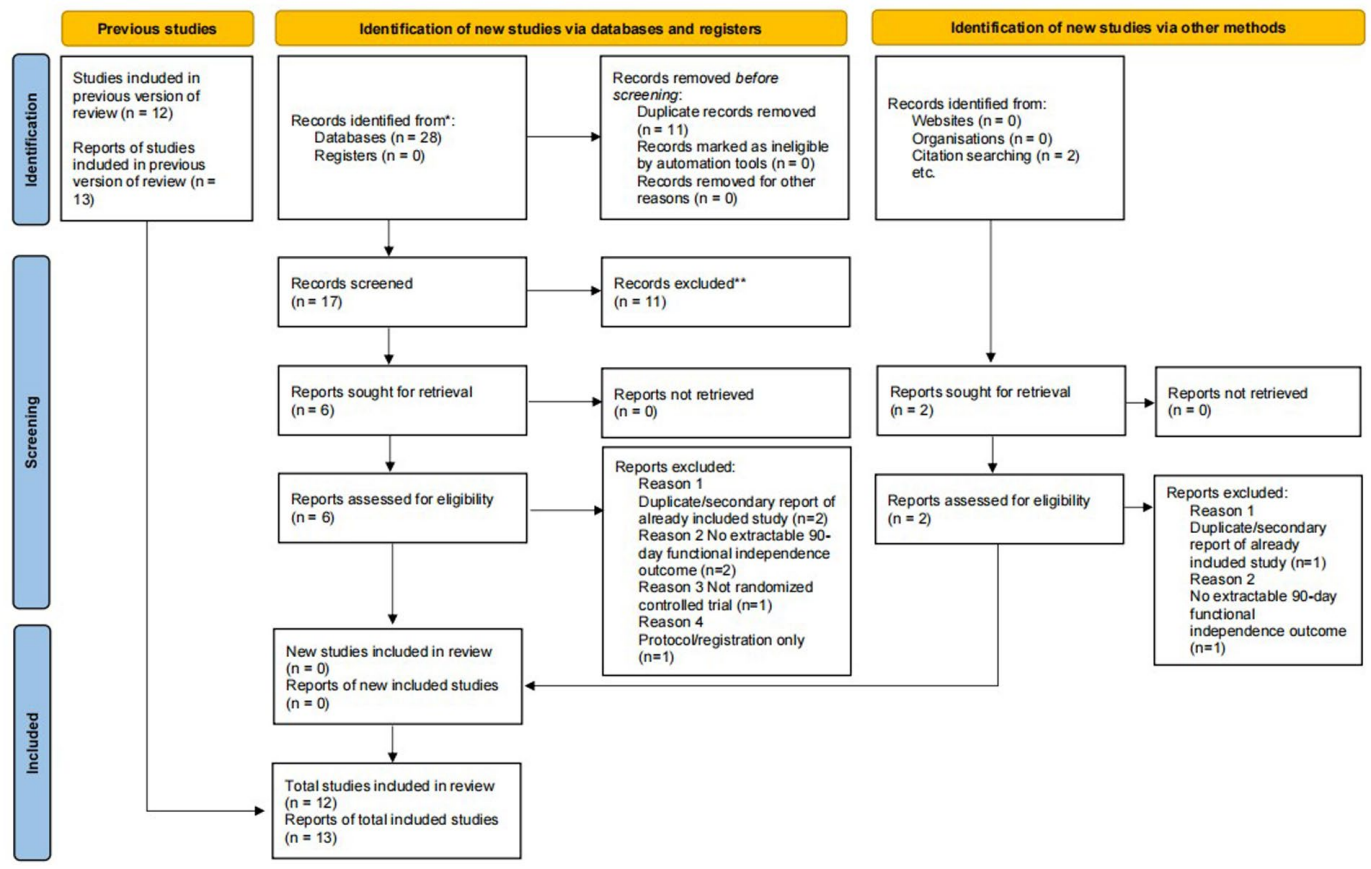
Updated PRISMA 2020 flow diagram showing the selection process of studies included in the previous version of the review and records identified in the updated search.

In the previous version of the review, 12 randomized controlled trials (RCTs) represented by 13 reports had been included. In the updated search, 28 additional records were identified from databases, with no additional records identified from registers. After removal of 11 duplicate records, 17 records were screened, of which 11 were excluded at the title and abstract stage. Six reports were sought for retrieval, all of which were successfully obtained and assessed for eligibility. These six reports were excluded for the following reasons: duplicate or secondary report of an already included study (*n* = 2), no extractable 90-day functional independence outcome (*n* = 2), not a randomized controlled trial (*n* = 1), and protocol/registration only (*n* = 1). Through citation searching, two additional reports were identified and assessed for eligibility; both were excluded, including one duplicate or secondary report of an already included study and one report without an extractable 90-day functional independence outcome. Therefore, no new eligible studies were added in the updated search. The final review remained unchanged, including 12 RCTs (13 reports) involving 5,301 patients. The detailed screening process is presented in Figure 1.

### Study characteristics and quality

The baseline characteristics of the included studies are summarized in Table 1. The included trials were published between 2014 and 2025, comprising 12 studies conducted in China, Denmark, the UK, France, and Romania. Sample sizes ranged from 26 to 1893 participants. Among them, 7 trials utilized a longer-duration RIC protocol (≥5 days), including the SERIC-EVT trial (9), the SERIC-IVT trial (10), RICAMIS (6), and studies by An et al. (17), He et al. (16), Che et al. (18), Poalelungi et al. (19). Five trials employed a shorter-duration protocol, including Blauenfeldt (RESIST) (7, 8, 20–23). Regarding reperfusion therapy, specific data for EVT patients were extracted from the SERIC-EVT trial (9) and the RESIST post-hoc analysis (8).

**Table 1:** Baseline characteristics of the included randomized controlled trials.

Study (year)	Country	Sample size (RIC/Control)	Mean age, y (RIC/Control)	Female, *n* (%)	Reperfusion therapy	RIC protocol	Duration
Guo et al., (9, 10) (EVT)	China	239/233	66.8/67.5	199/472 (42.2%)	100% EVT	5 × 5 min	Longer-duration
Guo et al. (9, 10) (IVT)	China	268/264	63.5/64.2	170/532 (32.0%)	100% IVT	5 × 5 min	Longer-duration
Chen et al. (6)	China	922/971	65.2/65.6	640/1893 (33.8%)	Mixed (mostly IVT)	5 × 5 min	Longer-duration
An et al. (17)	China	33/35	61.2/60.5	20/68 (29.4%)	Standard care	5 × 5 min	Longer-duration
He et al. (16)	China	24/25	67.2/66.5	15/49 (30.6%)	Standard care	5 × 5 min	Longer-duration
Che et al. (18)	China	15/15	62.4/64.1	9/30 (30.0%)	Standard care	5 × 5 min	Longer-duration
Poalelungi et al. (19)	Romania	20/20	65.3/63.8	16/40 (40.0%)	Standard care	5 × 5 min	Longer-duration
Blauenfeldt et al. (7)	Denmark	749/751	71.0/71.0	660/1500 (44.0%)	Mixed (IVT/EVT)	5 × 5 min	Shorter-duration
Pico et al. (23)	France	93/95	71.6/70.8	85/188 (45.2%)	Mixed (IVT/EVT)	5 × 5 min	Shorter-duration
England et al. (21)	UK	31/29	75.0/72.0	27/60 (45.0%)	100% IVT	4 × 5 min	Shorter-duration
England et al. (20)	UK	13/13	74.0/76.0	11/26 (42.3%)	100% IVT	4 × 5 min	Shorter-duration
Hougaard et al. (22)	Denmark	247/196	69.1/70.0	204/443 (46.0%)	Mixed (IVT/EVT)	4 × 5 min	Shorter-duration

RIC, remote ischemic conditioning; EVT, endovascular thrombectomy; IVT, intravenous thrombolysis.

Longer-duration was defined as repeated daily sessions for ≥5 days; shorter-duration was defined as a single session or treatment duration <5 days. Female proportion is presented for the total study population where reported. Mixed reperfusion therapy indicates studies including more than one reperfusion modality; “mostly IVT” indicates that intravenous thrombolysis was the predominant reperfusion strategy in that trial.

**Figure 2:**
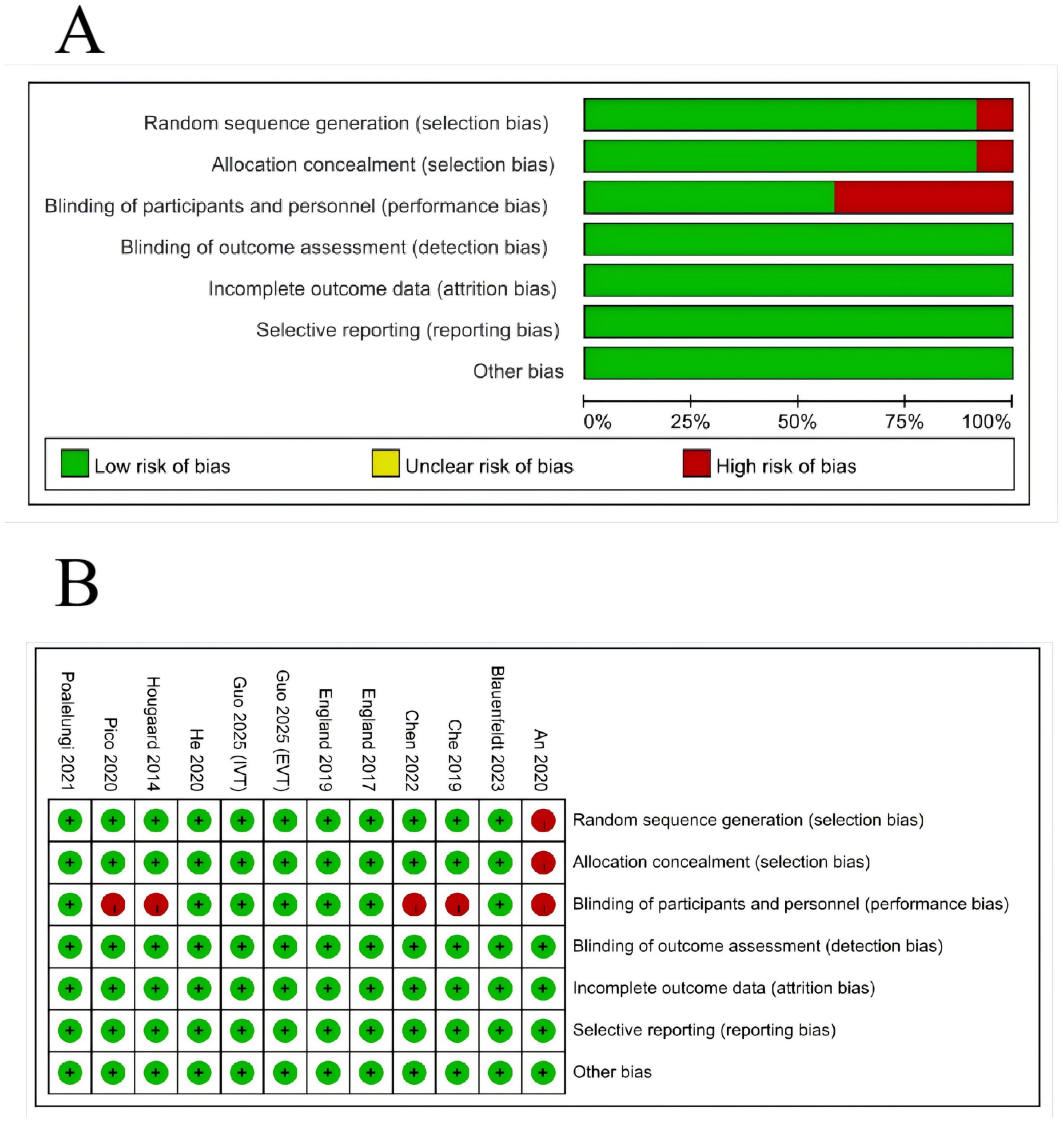
Risk of bias assessment. **(A)** Risk of bias graph presented as percentages across all included studies. **(B)** Risk of bias summary for each included study.

The risk of bias assessment is shown in Figure 2. Overall, the methodological quality of the included studies was moderate to high. Random sequence generation was adequately reported in most trials. Allocation concealment was generally low risk. Blinding of participants and personnel was assessed as high risk in the majority of studies due to the inherent difficulty of blinding the RIC intervention (lack of sham cuff in some protocols), whereas blinding of outcome assessment was low risk in all studies, ensuring the reliability of the mRS evaluation.

### Primary outcome: effect of treatment duration

**Figure 3:**
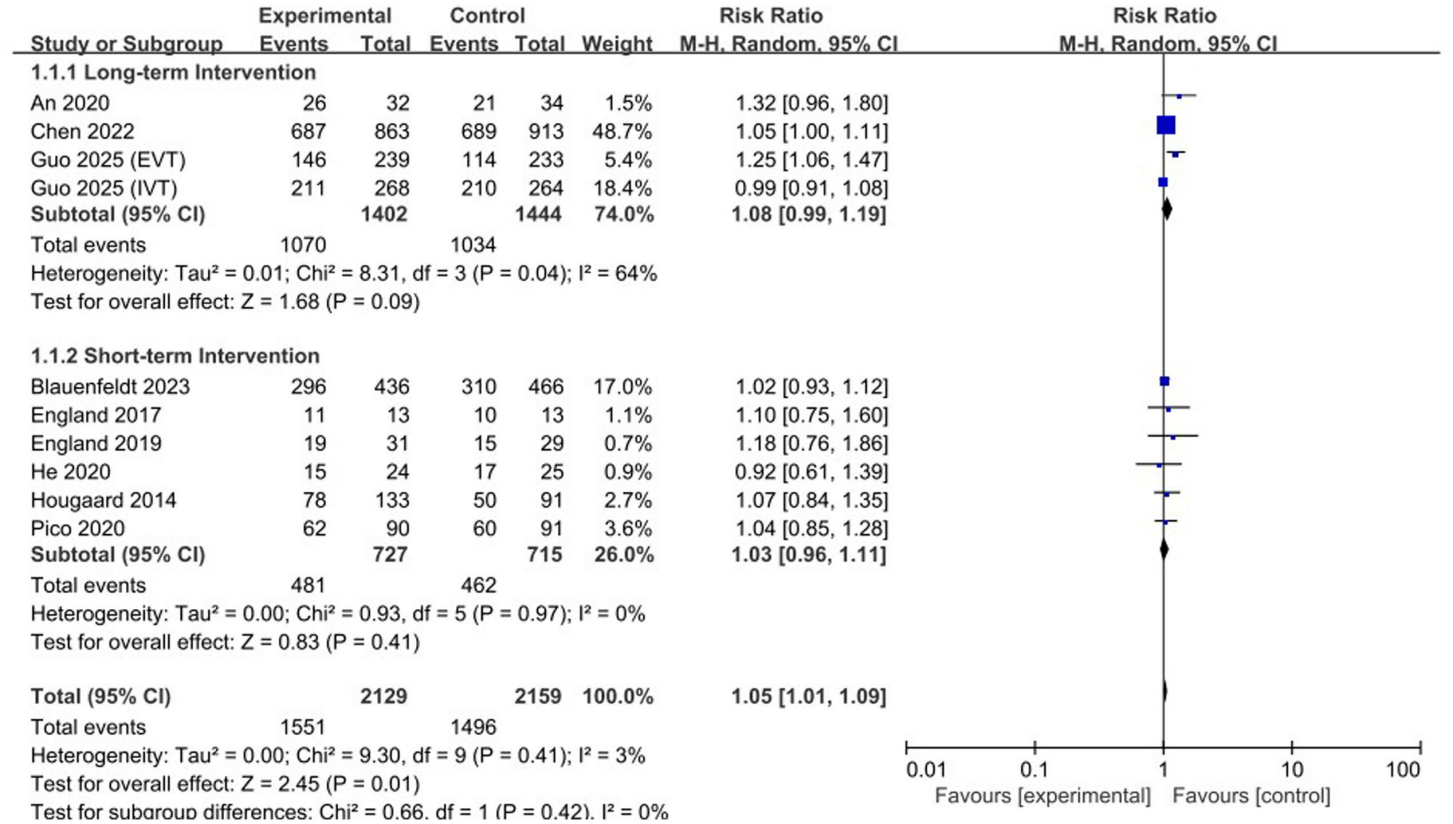
Forest plot of the primary outcome (functional independence at 90 days) stratified by treatment duration (longer-duration vs. shorter-duration).

The primary efficacy meta-analysis for functional independence at 90 days included 11 comparisons derived from the eligible RCT evidence base, because not all included trials provided extractable data for this endpoint. Overall, RIC was associated with a statistically significant increase in the rate of functional independence (mRS 0–2) at 90 days compared with the control group (RR 1.05, 95% CI 1.01–1.09, *p* = 0.01; Figure 3). The heterogeneity was low (*I*^2^ = 0%) (see Table 2).

**Table 2:** Analytic classification of the included randomized controlled trials according to treatment duration and reperfusion context.

Study (Year)	Country	Reperfusion context used in this review	RIC protocol per session	Treatment duration category used in this review	Main analytic contribution in this review
Guo et al. (9) (SERIC-EVT)	China	EVT	5 × 5 min	Longer-duration	Included in the EVT subgroup analysis; contributed to the evaluation of longer-duration RIC in thrombectomy-treated AIS
Guo et al. (10) (SERIC-IVT)	China	IVT	5 × 5 min	Longer-duration	Included in the IVT subgroup analysis; contributed to the evaluation of longer-duration RIC in thrombolysis-treated AIS
Chen et al. (6) (RICAMIS)	China	Mixed (mostly IVT)	5 × 5 min	Longer-duration	Major contributor to the overall pooled estimate; informed the evaluation of repeated RIC in a contemporary AIS population
An et al. (17)	China	Standard care	5 × 5 min	Longer-duration	Contributed to the longer-duration subgroup and non-reperfusion/mixed standard-care analytic context
He et al. (16)	China	Standard care	5 × 5 min	Longer-duration	Contributed to the longer-duration subgroup and non-reperfusion/mixed standard-care analytic context
Che et al. (18)	China	Standard care	5 × 5 min	Longer-duration	Contributed to the longer-duration subgroup and non-reperfusion/mixed standard-care analytic context
Poalelungi et al. (19)	Romania	Standard care	5 × 5 min	Longer-duration	Contributed to the longer-duration subgroup and non-reperfusion/mixed standard-care analytic context
Blauenfeldt et al. (7) (RESIST)	Denmark	Mixed (IVT/EVT)	5 × 5 min	Shorter-duration	Contributed to the shorter-duration subgroup; the parent trial also informed the later EVT *post hoc* analytic context
Pico et al. (23)	France	Mixed (IVT/EVT)	5 × 5 min	Shorter-duration	Contributed to the shorter-duration subgroup and mixed reperfusion analytic context
England et al. (21)	UK	IVT	4 × 5 min	Shorter-duration	Included in the IVT subgroup analysis; contributed to the evaluation of shorter-duration RIC in thrombolysis-treated AIS
England et al. (20)	UK	IVT	4 × 5 min	Shorter-duration	Included in the IVT subgroup analysis; contributed to the evaluation of shorter-duration RIC in thrombolysis-treated AIS
Hougaard et al. (22)	Denmark	Mixed (IVT/EVT)	4 × 5 min	Shorter-duration	Contributed to the shorter-duration subgroup and mixed reperfusion analytic context

AIS, acute ischemic stroke; EVT, endovascular thrombectomy; IVT, intravenous thrombolysis; RIC, remote ischemic conditioning.

Treatment duration categories were defined a priori in this review as longer-duration (repeated daily sessions for ≥5 days) and shorter-duration (single session or treatment duration <5 days), consistent with the subgroup framework described in the Methods. “Mixed” indicates trials enrolling participants across more than one reperfusion pathway. “Non-reperfusion/mixed standard-care analytic context” refers to studies without a pure EVT-only or IVT-only design that informed the subgroup comparison outside the EVT and IVT strata. This table is intended to clarify the analytic role of each included trial and to complement Table 1 rather than duplicate baseline demographic characteristics.

Exploratory subgroup analysis stratified by treatment duration suggested a possible difference in effect. The longer-duration RIC group (treatment duration ≥5 days) showed a trend toward improved functional outcomes (RR 1.08, 95% CI 0.99–1.19), whereas the shorter-duration RIC group (single session or <5 days) showed no clear signal of benefit (RR 1.03, 95% CI 0.97–1.09). However, these subgroup findings were not supported by formal interaction testing and should therefore be interpreted cautiously as hypothesis-generating rather than confirmatory.

### Exploratory subgroup analysis by reperfusion strategy

**Figure 4:**
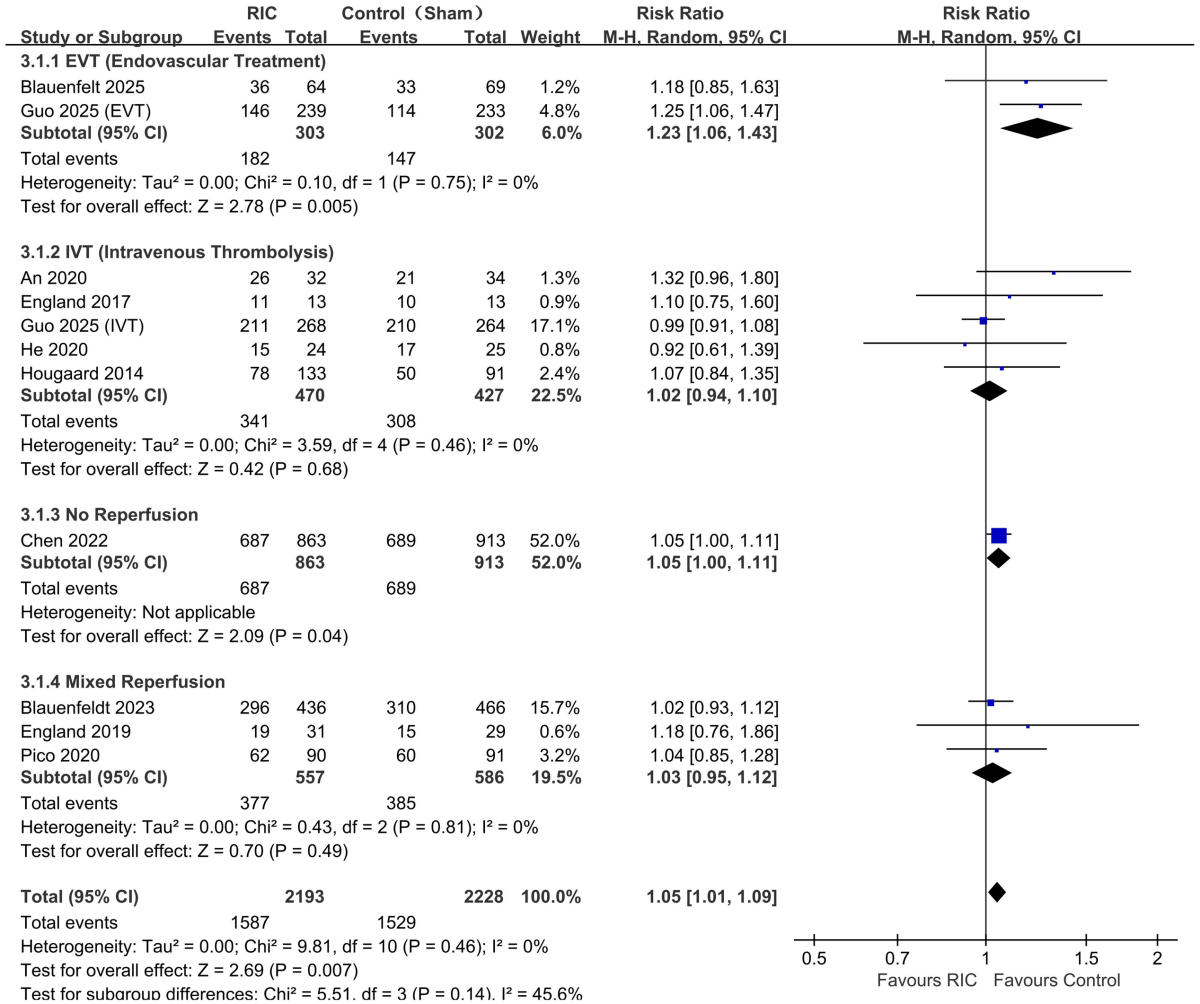
Forest plot of the primary outcome (functional independence at 90 days) stratified by reperfusion strategy (EVT, IVT, and non-reperfusion/mixed standard-care contexts).

To explore whether treatment effects differed across reperfusion contexts, we performed exploratory subgroup analyses according to reperfusion strategy (Figure 4).

RIC + EVT: In patients undergoing Endovascular Thrombectomy (EVT), a potential signal of benefit was observed for functional independence (RR 1.25, 95% CI 1.07–1.46, *p* = 0.005).

RIC + IVT: In contrast, among patients receiving exclusively Intravenous Thrombolysis (IVT), no additional benefit was observed (RR 0.99, 95% CI 0.93–1.06, *p* = 0.82), consistent with the neutral findings of the SERIC-IVT (10) and RECAST (21) trials.

No reperfusion: In the subgroup of patients who did not receive reperfusion therapy (or mixed standard care), a possible benefit was also observed (RR 1.40, 95% CI 1.01–1.93, *p* = 0.04). However, these subgroup findings should be interpreted cautiously because they were based on trial-level comparisons and were not supported by formal interaction testing.

### Safety outcomes

**Figure 5:**
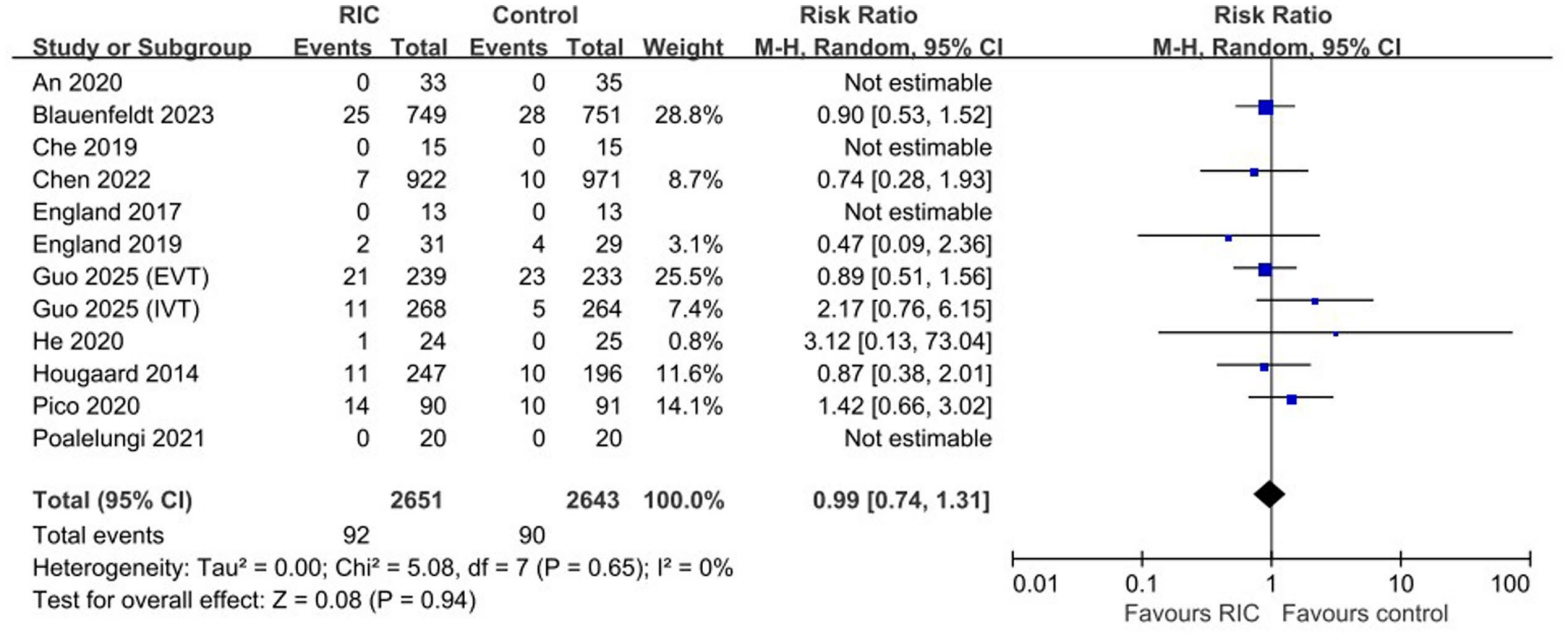
Forest plot of safety outcome (mortality at 90 days).

The safety of RIC was evaluated based on 90-day mortality data from 10 trials. RIC treatment did not increase the risk of mortality compared with the control group (RR 0.99, 95% CI 0.74–1.31, *p* = 0.94; Figure 5). No serious RIC-related adverse events (e.g., limb injury, skin necrosis, or intolerance) were reported in the included studies, confirming the high safety profile of the intervention even when applied for extended durations.

### Sensitivity analyses

Sensitivity analyses showed that the pooled estimate for the primary outcome was generally robust. After exclusion of He et al. (16), the pooled effect remained significant (RR 1.05, 95% CI 1.01–1.10). Similar results were observed after exclusion of the Blauenfeldt et al. (8) EVT sub-study report (RR 1.05, 95% CI 1.01–1.10) and in the strict dataset restricted to 90-day mRS 0–2 studies (RR 1.05, 95% CI 1.00–1.10). However, exclusion of the largest trial, Chen et al. (6), attenuated the pooled effect and rendered the association no longer statistically significant (RR 1.07, 95% CI 1.00–1.15), suggesting that the overall result was partly influenced by the largest study. Leave-one-out sensitivity analysis did not suggest major instability in the direction of the pooled effect. The corresponding leave-one-out and exclusion-based sensitivity-analysis forest plots are provided in the (Supplementary Figures S1–S5).

### Publication bias

**Figure 6:**
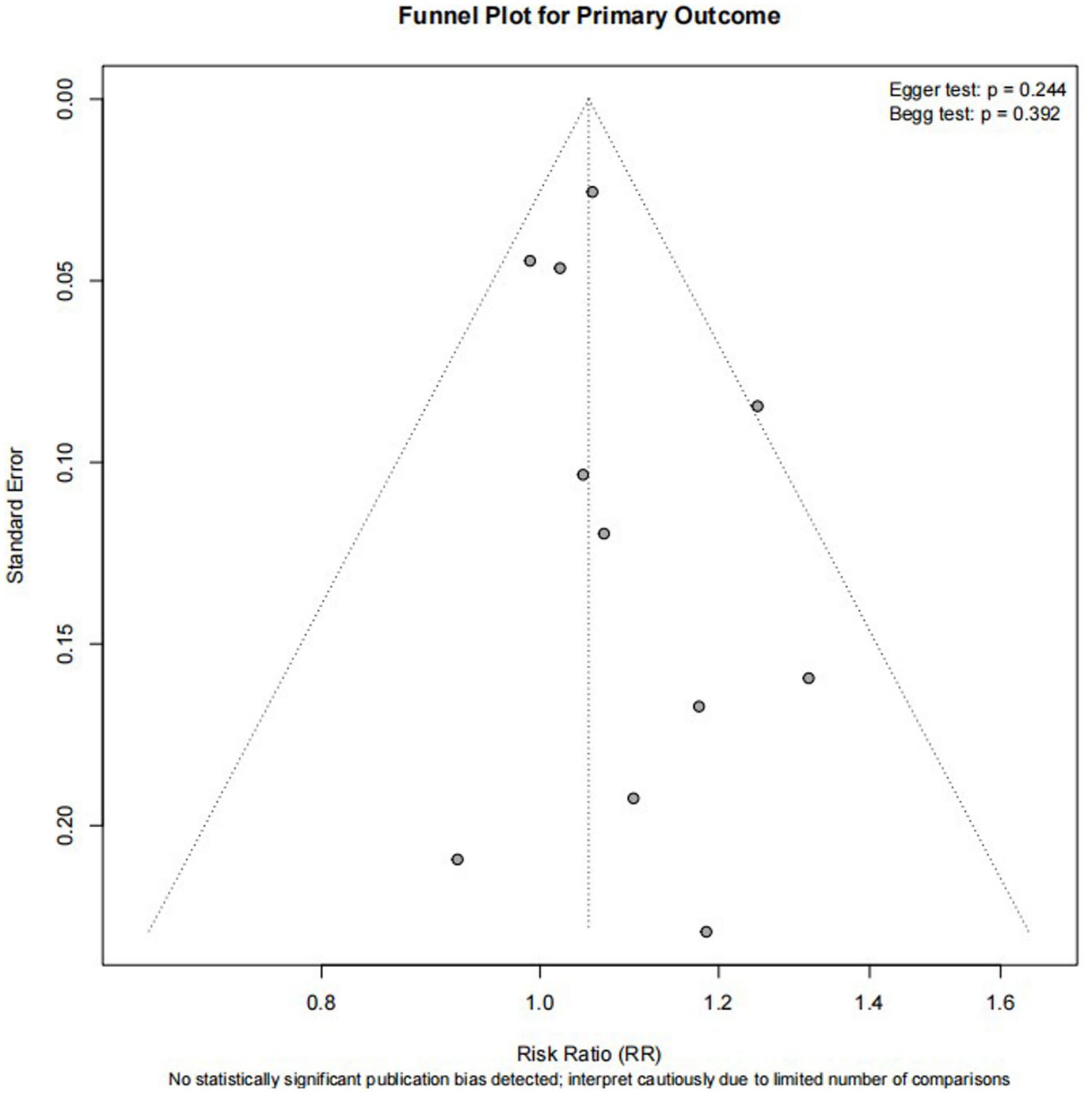
Funnel plot for the primary outcome (functional independence at 90 days). Formal assessment of small-study effects showed no significant funnel plot asymmetry (Egger’s test, *p* = 0.244; Begg’s test, *p* = 0.392).

Visual inspection of the funnel plot for the primary outcome (Figure 6) did not reveal marked asymmetry. In addition, Egger’s linear regression test did not indicate significant small-study effects (*p* = 0.244), and Begg’s rank correlation test was also non-significant (*p* = 0.392), suggesting that no statistically significant publication bias was detected. Nevertheless, these findings should be interpreted cautiously because the number of included comparisons was relatively limited, which may reduce the power of formal tests for publication bias.

## Discussion

### Summary of main findings

In this systematic review and meta-analysis incorporating the most recent high-quality RCT evidence identified through the final updated search in March 2026, we found that RIC was associated with a modest improvement in functional independence in patients with AIS. However, the magnitude of effect was small, and the clinical relevance of this pooled effect should be interpreted with appropriate caution. Our findings suggest that treatment duration and reperfusion status may partly explain the discrepant results of previous trials, but these effect-modifying roles cannot be considered definitive based on the currently available data. In particular, the most favorable signals were observed in studies using longer-duration protocols and in patients undergoing Endovascular Thrombectomy (EVT), whereas the intervention remained safe with no excess mortality. Sensitivity analyses were broadly consistent with the main result, although exclusion of the largest study attenuated the pooled effect and rendered the association no longer statistically significant, indicating that the overall estimate should be interpreted with caution.

### Comparison with previous meta-analyses

Our findings should be interpreted in the context of prior meta-analyses, particularly the comprehensive review by Kan et al. (38). That study pooled a broader set of randomized trials and ischemic cerebrovascular populations, including studies involving symptomatic intracranial arterial stenosis, transient ischemic attack, and carotid stenting-related populations, and concluded that chronic RIC could reduce stroke recurrence and improve prognosis, with bilateral upper-limb application, 5 cycles, and 50 min per session appearing optimal. In contrast, our review was designed to address a narrower and more contemporary clinical question by focusing on imaging-confirmed acute ischemic stroke and by incorporating pivotal recent RCTs published in 2025, including SERIC-EVT, SERIC-IVT, and the RESIST EVT *post hoc* analysis. Rather than attempting to redefine an “optimal” protocol, our analysis specifically examined whether treatment duration and reperfusion strategy may modify treatment effects in current AIS practice. Accordingly, our study should be viewed as an updated, AIS-focused, and clinically contextualized extension of the earlier literature rather than a simple duplication of prior work. Thus, the present study should be understood as addressing a more specific unresolved clinical question in contemporary AIS care, rather than merely repeating previous pooled analyses.

### The “dose” effect: Why duration matters?

The divergent outcomes between the neutral RESIST trial (7) and the positive RICAMIS trial (6) have sparked intense debate regarding the optimal “dose” of conditioning. Our subgroup analysis suggests that treatment duration may be one contributor to these discrepant findings, but the available evidence is insufficient to establish duration as a definitive determinant of efficacy. Because these analyses were based on between-trial subgroup comparisons rather than formal patient-level interaction testing, they should be regarded as exploratory and hypothesis-generating. Nevertheless, the observed pattern is biologically plausible. The neurovascular unit requires sustained support to recover from ischemic injury, a process that extends well beyond the hyperacute phase (24). Shorter-duration RIC may trigger immediate but transient protective mechanisms, whereas repeated RIC may exert more sustained effects on inflammation, vascular remodeling, and neuroplasticity (25–27). Accordingly, our findings should be interpreted as suggesting, rather than proving, that longer-duration RIC protocols may be more promising than single-session approaches.

### The synergistic effect: RIC and mechanical thrombectomy

A clinically interesting finding of our study was the apparent signal of benefit in the EVT subgroup (RR 1.25), whereas no corresponding signal was observed in the IVT subgroup (RR 0.99). However, this contrast should be interpreted cautiously. These subgroup findings were not based on direct head-to-head comparisons or formal interaction testing across all trials, and therefore they do not establish that EVT patients definitively benefit more from RIC than IVT-treated patients. Instead, they suggest a potentially important hypothesis for future study. One possible explanation relates to the completeness of recanalization. EVT often achieves more rapid and complete recanalization (Thrombolysis in Cerebral Infarction [TICI] grade 2b/3), which may improve delivery of humoral protective mediators to the ischemic penumbra (28). In contrast, IVT may result in partial or delayed recanalization, potentially limiting delivery of conditioning signals to the target tissue. In addition, RIC may attenuate ischemia–reperfusion injury (IRI) (29), a process that may be especially relevant when blood flow is abruptly restored to a large vascular territory, as in EVT (30). Thus, our findings raise the possibility that RIC may be more promising in EVT-treated patients, but this interpretation requires confirmation in adequately powered prospective trials.

### Clinical implications

Current guidelines do not routinely recommend RIC for acute stroke (1), and our findings do not justify a change in routine clinical practice at this stage. Instead, the present data suggest that RIC remains a potentially promising adjunctive strategy that warrants further refinement and prospective evaluation. In particular, future trials should clarify whether repeated longer-duration protocols are superior to single-session approaches and whether any benefit is concentrated in specific clinical contexts, such as EVT-treated patients or patients without effective reperfusion options (31). With the expanding indications for thrombectomy to larger ischemic cores and later time windows (32, 33), a low-cost and scalable adjunctive intervention such as RIC remains of interest, but its role should be considered investigational rather than established. This rationale is supported by mechanistic evidence for neural signaling in RIC and neurovascular-unit injury/repair, as well as by the broader clinical context of extended-window and stent-retriever thrombectomy (34–37).

### Strengths and limitations

The strengths of this study include the incorporation of the latest RCT data through 2025, enabling a timely update of the evidence base in acute ischemic stroke, and a clinically focused comparison of treatment duration and reperfusion strategy in a more restricted AIS population than that included in some previous meta-analyses. However, our study also has limitations. First, this review was not prospectively registered in PROSPERO, which may have reduced methodological transparency compared with prospectively registered systematic reviews. Second, blinding of participants is difficult in RIC trials (except for RESIST, which used a sham cuff), introducing a potential risk of performance bias, although outcome assessment was blinded in all studies. Third, there was heterogeneity in stroke severity, onset-to-treatment time, and background reperfusion management across the included trials. Fourth, the subgroup analyses by treatment duration and reperfusion strategy were exploratory, based on trial-level comparisons, and should not be interpreted as definitive evidence of effect modification. Fifth, although visual inspection of the funnel plot and formal statistical tests (Egger’s and Begg’s tests) did not indicate significant publication bias, the number of included comparisons remained relatively limited, and small-study effects therefore cannot be excluded with certainty. Sixth, sensitivity analyses were generally consistent; however, exclusion of the largest study attenuated the pooled effect and rendered it non-significant, suggesting that the overall estimate was partly influenced by the largest available trial. Seventh, although we excluded patients with peripheral arterial disease (PAD) to improve clinical homogeneity, the interaction between comorbidities and RIC efficacy requires further investigation.

## Conclusion

Remote ischemic conditioning appears to be a safe and potentially useful adjunctive therapy for acute ischemic stroke. In the current evidence base, any overall functional benefit appears modest, and the subgroup patterns observed for treatment duration and mechanical thrombectomy should be interpreted as suggestive rather than definitive. Taken together, these findings support continued investigation of RIC in acute ischemic stroke, particularly in trials designed to clarify the role of repeated treatment and specific reperfusion contexts, rather than routine clinical implementation at present.

## Data Availability

The raw data supporting the conclusions of this article will be made available by the authors, without undue reservation.
