# Supplementary material for: Optimizing remote ischemic conditioning for acute ischemic stroke: a systematic review and meta-analysis of treatment duration and reperfusion strategies
Source: Front Neurol. 2026 May 19;17:1787078. doi: 10.3389/fneur.2026.1787078 (PMC13225948; doi:10.3389/fneur.2026.1787078)

**Supplementary Figures: Sensitivity-analysis forest plots for the primary outcome**

Supplementary Figure S1. Leave-one-out sensitivity analysis for the primary outcome (functional independence at 90 days).


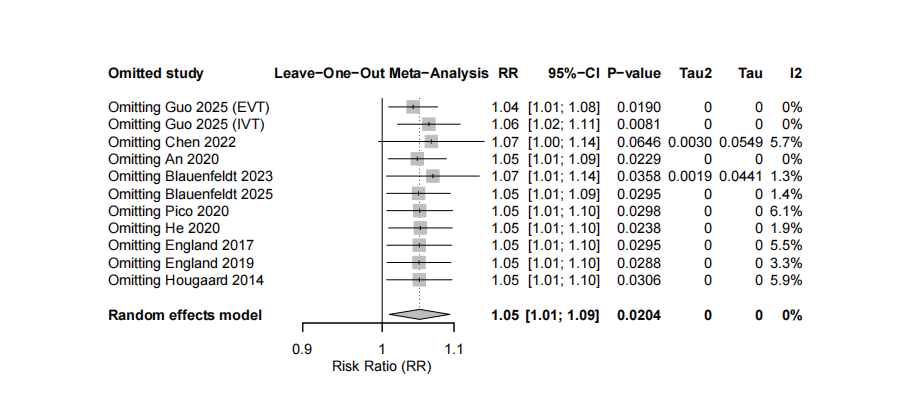


Supplementary Figure S2. Sensitivity analysis excluding He et al. (2020) for the primary outcome (functional independence at 90 days).


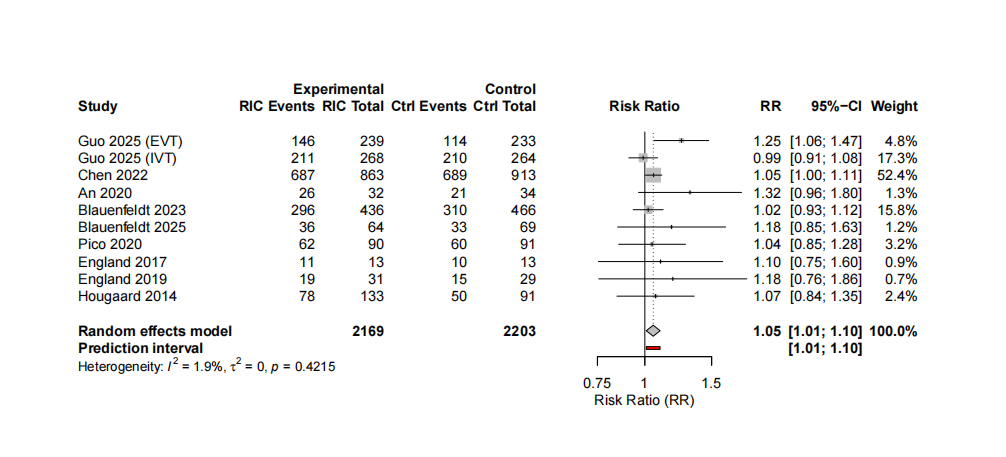


Supplementary Figure S3. Sensitivity analysis excluding Blauenfeldt et al. (2025) for the primary outcome(functional independence at 90days).


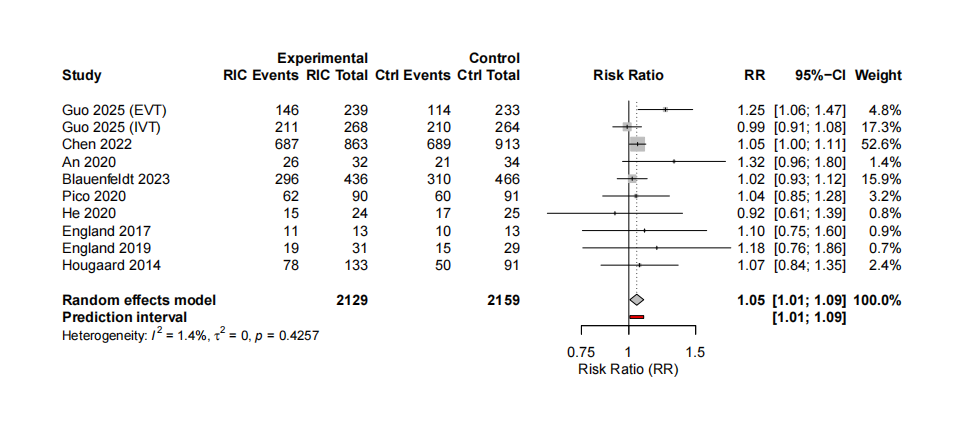


Supplementary Figure S4. Sensitivity analysis excluding Chen et al. (2022) for the primary outcome (functional independence at 90 days).


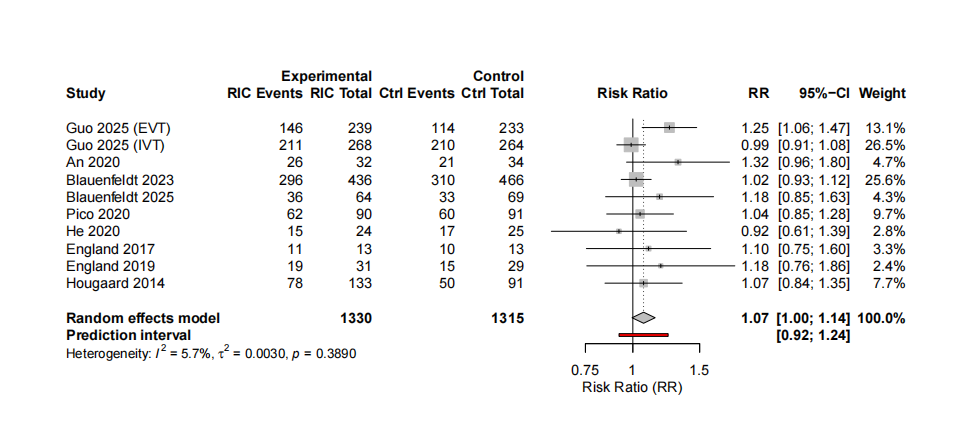


Supplementary Figure S5. Sensitivity analysis restricted to studies reporting extractable 90-day mRS 0–2 outcomes for the primary outcome (functional independence at 90 days).


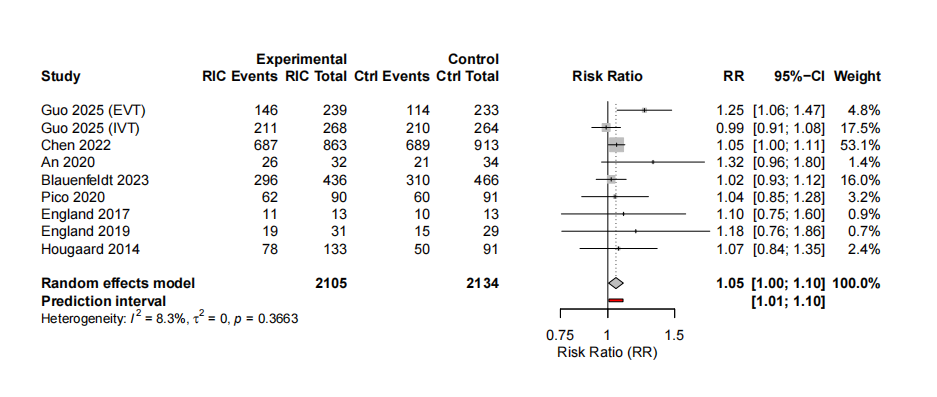

Supplement: Supplementary file 1 [file Supplementary_file_1.docx]
